# Biomarkers of stress in caregivers of children with special health care needs: A protocol for systematic review

**DOI:** 10.1097/MD.0000000000031448

**Published:** 2022-11-04

**Authors:** Luís Carlos Lopes-Júnior, Regina Aparecida Garcia Lima, Jaqueline Brosso Zonta, Mariane Caetano Sulino, Wendy Sue Looman, Leticia Mancini Correa, Aline Cristiane Cavicchioli Okido

**Affiliations:** a Graduate Program in Public Health, Health Sciences Center at the Federal University of Espírito Santo (UFES), Vitoria, ES, Brazil; b University of São Paulo at Ribeirão Preto College of Nursing (USP), Ribeirão Preto, SP, Brazil; c Health Sciences Center at the Federal University of São Carlos (UFSCar), São Carlos, SP, Brazil; d School of Nursing, University of Minnesota (UMN), Minneapolis, MN.

**Keywords:** Biomarkers, caregivers, Children with Special Health Care Needs, pediatrics, stress

## Abstract

Children with Special Health Care Needs (CSHCN) require greater attention from family caregivers as they present temporary or permanent physical, developmental, behavioral, or emotional issues. This demand for care generates overload and stress among family caregivers. So far, there is no study that gathers the available literature regarding the use of biomarkers to assess stress among caregivers of CSHCN. Hence, we aimed to synthetize and to assess the scientific evidence on biomarkers associated with stress in caregivers of CSHCN.

**Methods:**

This systematic review and meta-analysis protocol was elaborated following the Preferred Reporting Items for Systematic Reviews and Meta-Analyses Protocols (PRISMA-P). The search strategy will be undertaken through 7 electronic bibliographic databases: Embase, MEDLINE/PubMed, Cochrane Library, Web of Science, CINAHL, Scopus, and PsycINFO. In addition, secondary searches in other sources, such as Clinical trials.gov-NIH, The British Library, Pro Quest Dissertations Database, Google Scholar, and medRXiv will be also carried out. The reference section of the included studies will be hand searched for additional relevant studies. There will be no restriction regarding the publication dates or languages for this systematic review. Moreover, in an independently manner, 2 investigators will select studies, perform data extraction, as well as perform a critical appraisal of the risk of bias and overall quality of the selected studies, based on their designs. The heterogeneity among the studies will be assessed using the *I*^2^ statistic test. According to the results of this test, we will verify whether a meta-analysis is feasible. If feasibility is confirmed, a random-effect model analysis will be carried out. For data analysis, the calculation of the pooled effect estimates will consider a 95% CI and alpha will be set in 0.05 using the SPSS version 23.0.

**Results:**

This systematic review and meta-analysis will provide better insights regarding the biomarkers associated with stress in caregivers of CSHCN. Hence, consistent data and robust evidence will be provided to help practitioners and decision-makers in this area.

**Conclusions:**

To the best of our knowledge this study, will be the first to synthetize and critically evaluate the scientific evidence on biomarkers associated with stress in caregivers of CSHCN.

## 1. Introduction

Children with Special Health Care Needs (CSHCN) require continuous attention from family members and health professionals because they present physical, developmental, behavioral, or emotional problems of a temporary or permanent nature.^[1]^ Family caregivers perform actions that go beyond the care routinely provided to other children of the same age group, such as administering medication and enteral diets, glycemic control, psychomotor rehabilitation, oxygen therapy care, among others.^[2]^ The attention dedicated to childcare generates overload and stress among family caregivers, who are usually mothers.^[3]^ A recent systematic review that sought to synthesize existing research on the association between coping strategies and quality of life for caregivers of children with chronic illnesses suggested that greater complexity of care needs may be associated with worse caregiver well-being.^[4]^

Stress triggers emotional, behavioral problems and physical illnesses^[5,6]^ such as depression, anxiety, fatigue, sleep disturbances, and cognitive changes, negatively impacting the health-related quality of life of these caregivers.^[7,8]^ Most of the studies included in the systematic review and meta-analysis that evaluated the health outcomes of parents caring for children with chronic illnesses compared with parents of healthy children reported depression on the part of parents of children with chronic illnesses. The latter showed higher depression scores when compared with parents of healthy children (standardized mean difference = 0.35; 95% CI 0.26-0.35; *P* < .001).^[9]^

The stress suppresses important aspects of the immune response, such as the activity of Natural Killer cells and the proliferation of T cells.^[10]^ Exposure to a stressor, or stressors, activates the hypothalamic-pituitary-adrenal axis to release catecholamines through the sympathetic nervous system. Moreover, it activates neurons in the paraventricular nucleus of the hypothalamus, which secretes the corticotropin-releasing hormone. This hormone acts on the anterior pituitary promoting the release of the adrenocorticotropic hormone, which acts on the cortex of the adrenal gland, initiating the synthesis and release of glucocorticoids.^[10–12]^

Cortisol release follows a circadian rhythm, in which this hormone is normally secreted by the adrenal gland in short bursts, with 15 to 30 pulses over a day.^[10]^ Peripheral cortisol levels, reflect the activity of the hypothalamic-pituitary-adrenal axis and can be used as a biomarker to assess responses to stressful stimuli.^[11]^ High concentrations of cortisol inhibit antigen presentation, lead to atrophy of the lymphoid tissue of the thymus, spleen, and lymph nodes, besides stimulating apoptosis of lymphocytes. Additionally, increased levels of the cortisol inhibit the synthesis and release of cytokines and other mediators of immune and inflammatory reactions.^[8,10,12]^ Cortisol, in high concentrations, also modifies the balance between the cellular immune response (Th1) and the humoral (Th2) response, towards the Th2 response.^[11–14]^ These changes are associated with a decrease in the cytotoxic activity of lymphocytes and Natural Killer cells. Disruption of the immune balance can also lead to a chronic cascade of events mediated by pro-inflammatory cytokines (IL-1β, IL-6, TNF-α, and IFN), resulting in stress feedback loop, depression, anxiety, fatigue, sleep disorders, cognitive alterations, and subsequent negative impact on quality of life.^[12–16]^ Briefly, stress, through the activation of the neuroimmunoendocrine axis, can negatively influence multiple biological pathways.^[12]^ For instance, one study well-matched controls have examined cortisol in 82 mothers of children with psychiatric or developmental disorders. Cortisol typically shows a robust daily pattern, rising shortly after awakening (the cortisol awakening response, CAR), and then gradually declining over the course of the day. The findings showed that mothers of children with psychiatric or developmental disabilities, however, had less pronounced daily declines in their diurnal patterns of cortisol, especially on days when they had increased contact with their children.^[17]^ Another research found that compared to mothers of typically developing children, mothers of children with autism spectrum disorders and other developmental disabilities mounted a poor antibody response to pneumococcal vaccinations, indicating a reduced capacity to ward off infections.^[18]^

Investigations analyzing the stress of caregivers of CSHCN from these biomarkers are increasing.^[19–21]^ However, there is still a gap in the literature regarding the synthesis of available evidence on the use of biomarkers to assess stress among caregivers of CSHCN. In this sense, the purpose of this systematic review is to synthetize and to assess the scientific evidence on biomarkers associated with stress in caregivers of CSHCN.

## 2. Methods

### 2.1. Design

This protocol was elaborated following Preferred Reporting Items for Systematic Reviews and Meta-Analyses Protocols.^[22]^ Registration of this SR protocol was obtained from the International Prospective Register of Systematic Reviews - PROSPERO (Registration ID: CRD42021258051). The systematic review and meta-analysis will also be reported based on the PRISMA 2020 statement.^[23]^

### 2.2. Search strategy

The search strategy will be conducted in 7 electronic bibliographic databases: EMBASE (Excerpta Medica dataBASE) (from 1973 to December 31, 2021), MEDLINE/PubMed (from 1947 to December 31, 2021), Cochrane Library (from 1991 to December 31, 2021), Web of Science (from 1985 to December 31, 2021), SCOPUS (from 2004 to December 31, 2021), and Cummulative Index to Nursing and Allied Health Literature (from 1961 to December 31, 2021) and PsycINFO (from 1967 to December 31, 2021). In addition, secondary searches in other sources, such as Clinical trials.gov-NIH, The British Library, Google Scholar and pre-prints for Health Sciences [medRXiv] will be also carried out. The reference section of the included studies will be hand searched for additional relevant studies. No restriction regarding publication date, setting or language will be considered in this systematic review.

The PECOS (Population, Exposure/Comparison/Outcomes/Study Design) acronym^[24]^ was used to elaborate our research question (Table 1). Using the PECOS approach we elaborated the following research question of this review: “What scientific evidence are available about levels of stress-related biomarkers among Children with Special Health Care Needs” caregivers?

**Table 1:** PECOS acronym for search strategy.

PECOS component^[22]^	Inclusion criteria	Exclusion criteria
P—Population	Caregivers of Children with Special Health Care Needs (CSHCN)	Caregivers of Children who are not CSHCN
E—Exposure	Not applicable	–
C—Comparison	Not applicable	–
O—Outcome	Self-reported stress and level of biomarkers associated with stress	
S–Study Design	Quantitative studies	Qualitative studies

Initially, we will identify the existence of a specific subject heading index in each database (including Medical Subject Headings terms, Emtree terms, PsycINFO Thesaurus and DeCS-Health Science Descriptors) and related synonyms (keywords). The search terms will be combined using the Boolean operators “AND” and “OR.”^[25–28]^

The preliminary pilot search strategy combining Medical Subject Headings terms, synonyms (entry terms) as well as keywords that will be used in MEDLINE/PubMed is detailed in Table 2. Two researchers will perform the search strategy simultaneously in all databases. The bibliographic software EndNote will be used to store, organize, and manage the overall references and ensure a systematic and comprehensive search.

**Table 2:** Preliminary search strategy in the MEDLINE via PubMed.

MEDLINE/PubMed	Search strategy
	**#1** ((“Caregivers” [MeSH Terms] OR “Caregiver” [All Fields] OR “Caregiver, Family” [All Fields] OR “Family Caregiver” [All Fields] OR “Informal Caregivers” [All Fields] OR “Mothers” [All Fields] OR “Parents” [All Fields] OR “Caregiver Burden” [MeSH terms] OR “Burden, Caregiver” [All Fields] OR “Caregiver Exhaustion” [All Fields]))
	**#2** ((“Disabled Children” [MeSH Terms] OR “Children with Disability” [All Fields] OR “Children, Disabled” [All Fields] OR “Handicapped Children” [All Fields] OR “Children, Handicapped” [All Fields] OR “Child, Disabled” [All Fields] OR “Disabled Child” [All Fields] OR “Medically Fragile Children” [All Fields] OR “Children with Medical Complexity” [All Fields] OR “Technology-dependent Children” [All Fields] OR “Children with Complex Chronic Conditions” [All Fields]))
	**#3** #1 AND #2
	**#4** ((“Stress, Physiological” [MeSH Terms] OR “Stress” [All Fields] OR “Biomarkers” [MeSH Terms] OR “Marker, Biological” [All Fields] OR “Biological Marker” [All Fields] OR “Biologic Marker” [All Fields] OR “Biomarker” [All Fields] OR “Immune Markers” [All Fields] OR “Markers, Immune” [All Fields] OR “Serum Markers” [All Fields] OR “Markers, Serum” [All Fields] OR “Clinical Markers” [All Fields] OR “Biochemical Marker” [All Fields] OR “Markers, Biochemical” [All Fields] OR “Marker, Biochemical” [All Fields] OR “Glucocorticoids” [MeSH terms] OR “Glucocorticoid” [All Fields] OR “Glucocorticoid Effect” [All Fields] OR “Effect, Glucocorticoid” [All Fields] OR “Glucorticoid Effects” [All Fields] OR “Receptors, Glucocorticoid” [MeSH terms] OR “Glucocorticoid Receptors” [All Fields] OR “Receptors, Glucocorticoids” [All Fields] OR “Glucocorticoid Receptor” [All Fields] OR “Receptor, Glucocorticoid” [All Fields] OR “Cortisol” [All Fields] OR “Salivary Cortisol” [All Fields] OR “Alpha-Amylases” [MeSH terms] OR “Alpha Amylases” [All Fields] OR “Alpha-Amylase” [All Fields] OR “Salivary Alpha-Amylases” [MeSH terms] OR “Salivary Alpha-Amylase” [All Fields] OR “Salivary alpha Amylase” [All Fields] OR “Alpha-Amylase, Salivary” [All Fields]))
	**#5 #3 AND #4**

### 2.3. Eligibility criteria

In this systematic review, we will include the overall observational as well as experimental study designs, i.e., cross-sectional, cohort, case-control, ecological, descriptive, and clinical trials. Handsearching will be carried out in the reference lists and in the gray literature seeking additional studies. Studies comprising caregivers of children aged 2 to 12 years will be included, according to the Medical Subject Headings term “Child.” Moreover, language and date restrictions will not be employed in the search strategy.

In order to make sure that the children participating in the study are characterized as CSHCN, the Children with Special Health Care Needs Screener (CSHCN Screener) will be used, an instrument originated in the USA capable of identifying and evaluating the care demands of children in 3 domains: dependence on drugs prescribed for a certain clinical condition, use of health services above what is considered normal or routine, and the presence of functional limitations. The presence of one or more domains characterizes it as CSHCN.^[29]^

Additionally, potential markers of stress include thermal stress markers, such as heat shock proteins, innate immune markers, such as acute phase proteins, oxidative stress markers, and chemical secretions in the saliva and urine.^[30]^

### 2.4. Study selection

Firstly, duplicates retrieved in all databases will be removed using the Endnote program. The screening will be carried out independently by 2 researchers (ACCO and LCLJ) based on titles and abstracts of the studies, on the meeting of the pre-established inclusion and exclusion criteria, and on the blind use of the Rayyan application, developed by the Qatar Computing Research Institute as an auxiliary tool in the archiving, organization, and selection of articles.

**Figure 1.**
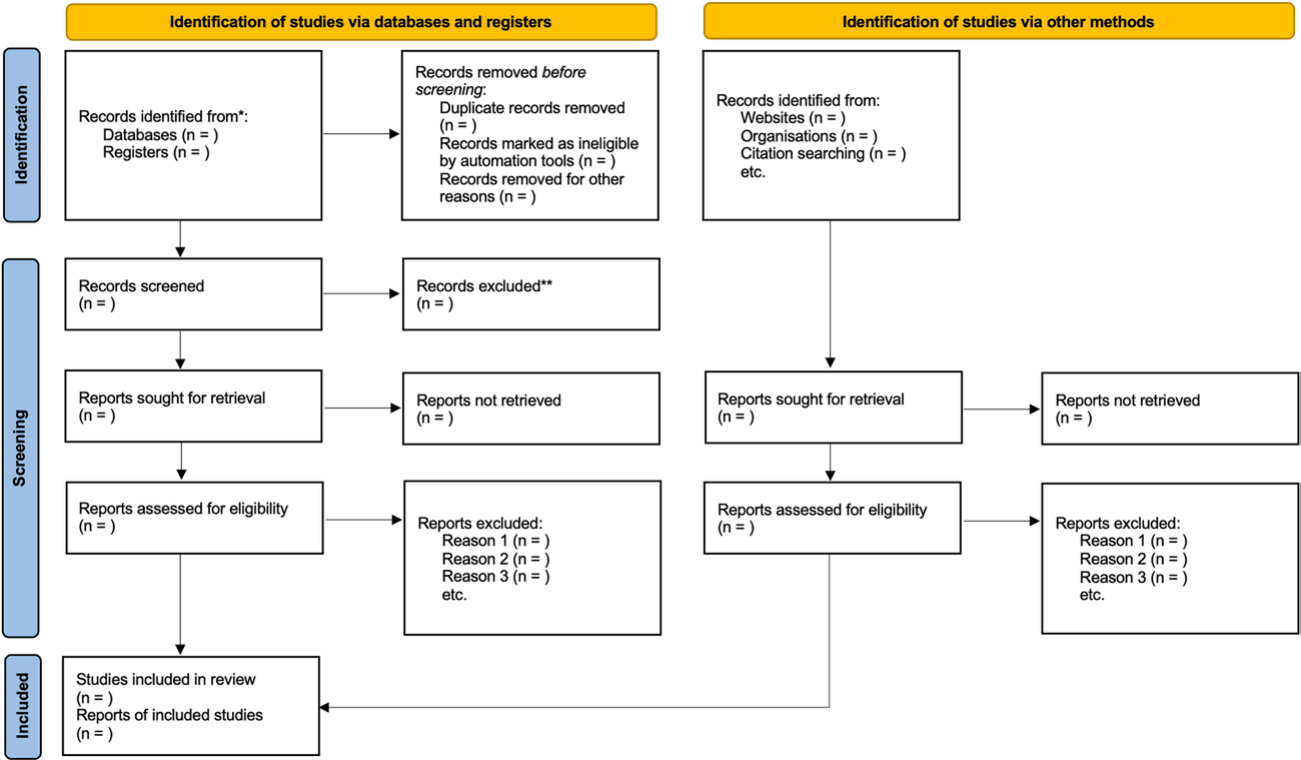
PRISMA flow diagram.

The same 2 reviewers (ACCO and LCLJ) will assess the full text of the retrieved articles independently to check whether they meet all the inclusion criteria. Discrepancies between the reviewers will be resolved either by discussion or, in the lack of agreement, by a third reviewer (RAGL) using the Rayyan app (http://rayyan.qcri.org) again. A flowchart will summarize the study selection process in line with the PRISMA 2020 statement^[23]^ (Fig. 1).

### 2.5. Data extraction

Two researchers (ACCO and LCLJ) will independently perform data extraction for each included study based on forms published previously.^[25,28,31–33]^ Information to be extracted includes identification of the study and objectives; study population and baseline characteristics; type of exposure; study methodology; recruitment methods; biomarkers assessed; times of measurement; follow-up; outcomes; main findings; clinical and epidemiological significance; and conclusions. Supplementary Table 1, http://links.lww.com/MD/H782 shows the standardized form created for data extraction.

### 2.6. Critical appraisal

The internal validity and risk of bias for observational studies will be assessed according to the study design. For instance, the Newcastle-Ottawa Scale will be used to evaluate internal validity of cohort studies as well as case-control study design.^[34]^ The JBI Critical Appraisal Checklist for Prevalence Data will be used for assessing cross-sectional studies.^[35]^ To evaluate risk of bias in randomized controlled trials the RoB 2 will be used.^[36]^ The same 2 reviewers (ACCO and LCLJ) will perform the critical appraisal independently.

### 2.7. Data synthesis

Study characteristics will be presented in tables. Heterogeneity among studies will be measured by the *I*^2^ statistic to estimate the percentage of variation across studies, ranging from 0% to 100%,^[37,38]^ and its interpretation is low heterogeneity when *I*^2 ^= 0%–40%; moderate heterogeneity when *I*^2 ^= 30%–60%; substantial heterogeneity when *I*^2 ^= 50%–90% represents a; and high heterogeneity when *I*^2 ^= 75%–100%.^[28,39,40]^

Furthermore, the subgroup analysis will be performed using a random-effect model analysis adjusted for age and sex. In addition, it will be carried out to explore the heterogeneity across studies. According to the *I*^2^ statistic, we will determine whether a meta-analysis is feasible.^[25,40,41]^ For data analysis, the calculation of the pooled effect estimates will consider a 95% CI and alpha set in 0.05 using the SPSS version 23.0. Besides, we will rate the certainty of evidence based on Cochrane methods and in accordance with the Grading of Recommendations Assessment, Development and Evaluation (GRADE).^[42]^ The evaluation of the quality of evidence in the evaluated studies will be independently performed in a paired manner by 2 reviewers (ACCO and LCLJ). Disagreements will be addressed by a third reviewer (RAGL).

### 2.8. Ethical aspects and dissemination plans

Because this is a systematic review protocol, and since our analysis will only include previously published data, the Institutional Review Board approval was not applicable. Moreover, the systematic review and meta-analysis will be performed in accordance with the PRISMA 2020 statement.^[23]^ Additionally, any amendments to this protocol will be registered. Regarding the dissemination plans, we intend to disseminate the results via peer-reviewed publication and via presentations in international conferences.

## 3. Discussion

Two main potential limitations include the predominance of cross-sectional studies that might limit the generalizability of the results, mainly with regards to cause-and-effect relationship, given the study design; and the risk of type 1 error (arising from the bias in recruiting participants, confounding factors, subgroup, and sensitivity analyses of the potential studies). However, the systematic review will provide an up-to-date synthesis regarding the biomarkers associated with stress in caregivers of CSHCN. Hence, consistent data and robust evidence will be provided to help practitioners and decision makers in this area.

The results of this review may support more specific and personalized nursing interventions based on the circadian pattern that is presented, and generate rich opportunities for future intervention research.^[13,43,44]^ By elucidating the behavior of these biomarkers among CSHCN caregivers, it will be possible to identify, for example, critical moments of the day when the most abrupt changes in these biomarkers occur, as well as to identify whether the type of care required by the child influences or not the circadian pattern of the child’s life. For example, are changes in the first measurement of the day associated with sleep disturbances in these caregivers? Do caregivers of children who remain bedridden and who require technological devices to maintain life (e.g., mechanical ventilation) have greater impairment of the caregivers’ circadian rhythm pattern?

## 4. Conclusion

This systematic review and meta-analysis, to the best of our knowledge, will be the first to synthetize and critically evaluate the scientific evidence on biomarkers associated with stress in caregivers of Children with Special Health Care Needs. Finally, we believe that this study will provide consistent evidence that will aid the practitioners make decisions in clinical practice.

## Author contributions

LCLJ and ACCO conceived the idea and planned and designed the study protocol. LCLJ and ACCO wrote the first draft. LCLJ, RAGL, JBZ, WSL, LMC, and ACCO planned the data extraction and statistical analysis. LCLJ, RAGL, JBZ, WSL, LMC, and ACCO provided critical insights. LCLJ and ACCO critically reviewed and modified the manuscript. All authors have reviewed and approved the manuscript. LCLJ and ACCO are responsible for the overall content as guarantor.

**Conceptualization:** Luís Carlos Lopes-Júnior, Aline Cristiane Cavicchioli Okido.

**Data curation:** Luís Carlos Lopes-Júnior, Regina Aparecida Garcia Lima, Jaqueline Brosso Zonta, Mariane Caetano Sulino, Wendy Sue Looman, Leticia Mancini Correa, Aline Cristiane Cavicchioli Okido.

**Formal analysis:** Luís Carlos Lopes-Júnior, Regina Aparecida Garcia Lima, Wendy Sue Looman, Aline Cristiane Cavicchioli Okido.

**Investigation:** Luís Carlos Lopes-Júnior, Aline Cristiane Cavicchioli Okido.

**Methodology:** Luís Carlos Lopes-Júnior.

**Project administration:** Aline Cristiane Cavicchioli Okido.

**Resources:** Aline Cristiane Cavicchioli Okido.

**Software:** Luís Carlos Lopes-Júnior, Aline Cristiane Cavicchioli Okido.

**Supervision:** Luís Carlos Lopes-Júnior, Aline Cristiane Cavicchioli Okido.

**Validation:** Luís Carlos Lopes-Júnior, Jaqueline Brosso Zonta, Wendy Sue Looman, Aline Cristiane Cavicchioli Okido.

**Visualization:** Luís Carlos Lopes-Júnior, Regina Aparecida Garcia Lima, Jaqueline Brosso Zonta, Mariane Caetano Sulino, Wendy Sue Looman, Aline Cristiane Cavicchioli Okido, Leticia Mancini Correa.

**Writing—original draft:** Luís Carlos Lopes-Júnior, Regina Aparecida Garcia Lima, Jaqueline Brosso Zonta, Mariane Caetano Sulino, Wendy Sue Looman, Leticia Mancini Correa, Aline Cristiane Cavicchioli Okido.

**Writing—review & editing:** Luís Carlos Lopes-Júnior, Regina Aparecida Garcia Lima, Aline Cristiane Cavicchioli Okido.

## Supplementary Material


