# Supplementary material for: Biomarkers of stress in caregivers of children with special health care needs: A protocol for systematic review
Source: Medicine (Baltimore). 2022 Nov 4;101(44):e31448. doi: 10.1097/MD.0000000000031448 (PMC9646662; doi:10.1097/MD.0000000000031448)
Supplement: Supplementary file 1 [file medi-101-e31448-s001.pdf]

Supplementary Table 1. Data extraction form.

Study number:	**Level of evidence:**<br>**Methodological Appraisal tool:**
**STUDY CARACTERISTICS**	
Authors	
Title	
Year of publication	
Country	
Conflicts of interests	
Sponsorship	
Background	
Rationale	
Hypothesis tested	
Objectives	
Methods	
Methodology is reported according with STROBE (observational studies) or CONSORT (experimental studies)<br>( ) Yes<br>( ) No<br>If yes, what was the degree of compliance of the checklist?<br>( ) High compliance<br>( ) Intermediate compliance<br>( ) Low compliance	
Study design	
Local:	
Sample size and calculation:	
Inclusion criteria (definition of exposure of interest)	
Exclusion criteria	
Confounding factors/Interaction factors considered	
Ethical aspects	
Procedure for data collection:<br>- Collection period:<br>- Procedures:	
Instruments for data collection for interest outcome<br>▪ What instrument was used?<br>▪ For scales, do higher values indicate worsening of stress?	

▪ For scales, what is the minimum value?<br>▪ For scales, what is the maximum value??	
Outcomes / Evaluation of outcomes<br>- Primary outcome:<br>- Secondary outcome:<br><br>**Continuous outcomes**<br>▪ Baseline<br>▪ Follow-up time<br>▪ Number of patients<br>▪ Mean (M)<br>▪ Standard Deviation (SD)<br>▪ Other precision and dispersion measurements (if necessary)<br>▪ Final score of instruments (validated scales)<br>▪ Level of the stress biomarker at baseline and in the end of study (M±SD)<br>▪ Total number of patients who experienced the outcome<br>▪ Number of patients who did not experience the outcome<br>▪ Circadian patterns of stress across the day	
Follow-up	
Statistical analysis	
If, cohort study	I. Number of participants in the exposed and unexposed cohort:<br>II. Number of participants in each group:<br>III. Comparability of exposed and unexposed cohorts<br>IV. Contamination (unexposed patient being exposed):<br>V. Follow-up period:<br>VI. Dropouts:
If, case-control study	I. Criteria for selection of cases:<br>II. Criteria for selection of controls:<br>III. Comparability of groups:<br>IV. Dropouts:
If, experimental or quase-experimental study	**a)** Trial Register:<br><br>**b)** Trial arms:<br>- Experimental Group:<br><br>**c)** Randomization:<br><br>**d)** Masking:<br><br>**e)** Intervention protocol:<br><br>**f)** Per-protocol and modified intention-to-treat analyses:<br>- Per-protocol:<br>- Intention-to-treat:

	- Dropouts:
Results	
Main results	
Clinical-Epidemiological Significance	
Limitations of the study	
Strengths of the study	
Conclusions	
Main conclusions	
Implication for clinical practice and research or for decision-makers / stakeholders	
